# Trans-fracture approach for ORIF of coronal shear fractures of the distal humerus

**DOI:** 10.1007/s00402-022-04501-6

**Published:** 2022-06-22

**Authors:** Michael Hackl, Fabian Lanzerath, Christian Ries, Andreas Harbrecht, Tim Leschinger, Kilian Wegmann, Lars Peter Müller

**Affiliations:** 1 Faculty of Medicine, University of Cologne, Cologne, Germany; 2 Center of Orthopedic and Trauma Surgery, University Hospital Cologne, Cologne, Germany; 3 Department of Orthopedics, University Medical Center Hamburg-Eppendorf, Hamburg, Germany

**Keywords:** Distal humerus, Fracture, Coronal shear fracture, Elbow, ORIF, Surgical approach

## Abstract

**Introduction:**

Open reduction and internal fixation (ORIF) of comminuted coronal shear fractures of the distal humerus is challenging. When a concomitant lateral condyle fracture is present, it may be used for a trans-fracture approach to facilitate exposure and fracture reduction. This study aimed to investigate the frequency of lateral condyle fractures in coronal shear fractures of the distal humerus and analyze fracture reduction, fracture union and clinical results following ORIF through a trans-fracture approach.

**Materials and methods:**

All adult patients who underwent treatment for an acute distal humerus fracture during a three-year period in our level-one trauma center were identified. All fractures were classified according to the Orthopaedic Trauma Association (OTA/AO) fracture classification system and all B3 fractures were classified according to the Dubberley classification. B3 fractures with a concomitant radial condyle fracture were identified. The clinical and radiological results, (Mayo Elbow Performance Score = MEPS, Visual Analogue Scale = VAS, range of motion), complications and revision surgeries were analyzed.

**Results:**

53 patients (mean age 52 ± 19 years) were identified. 13 fractures (24.5%) were B3 fractures. Four of them (30.8%) had a concomitant radial condyle fracture. All of these patients underwent ORIF with headless cannulated compression screws and a (postero-)lateral locking plate through a trans-fracture approach. At a minimum follow-up of 24 months, the MEPS was 88 ± 12 points, the VAS was 2 ± 1 and the range of motion was 118° ± 12°. All fractures showed anatomic reduction. One patient developed partial avascular necrosis and underwent arthrolysis at 6 months. One patient underwent partial hardware removal and lateral collateral ligament bracing at 6 months.

**Conclusions:**

Lateral condyle fractures are present in about one third of coronal shear fractures of the distal humerus. This injury can be used for a trans-fracture approach to facilitate exposure and to reliably achieve anatomic fracture reduction.

## Introduction

Coronal shear fractures of the distal humerus are among the most complex elbow pathologies. Hence, postoperative complications—such as non-union, avascular necrosis, stiffness, painful osteoarthritis, instability and ulnar neuropathy—are frequently observed [1–9]. Anatomic reduction of the articular surface is crucial to increase the chances of a good clinical outcomes. To reduce the fracture anatomically, sufficient exposure is key but can be difficult to obtain when trying to preserve the blood supply of the fragments and respecting the integrity of the collateral ligaments.

**Fig. 1:**
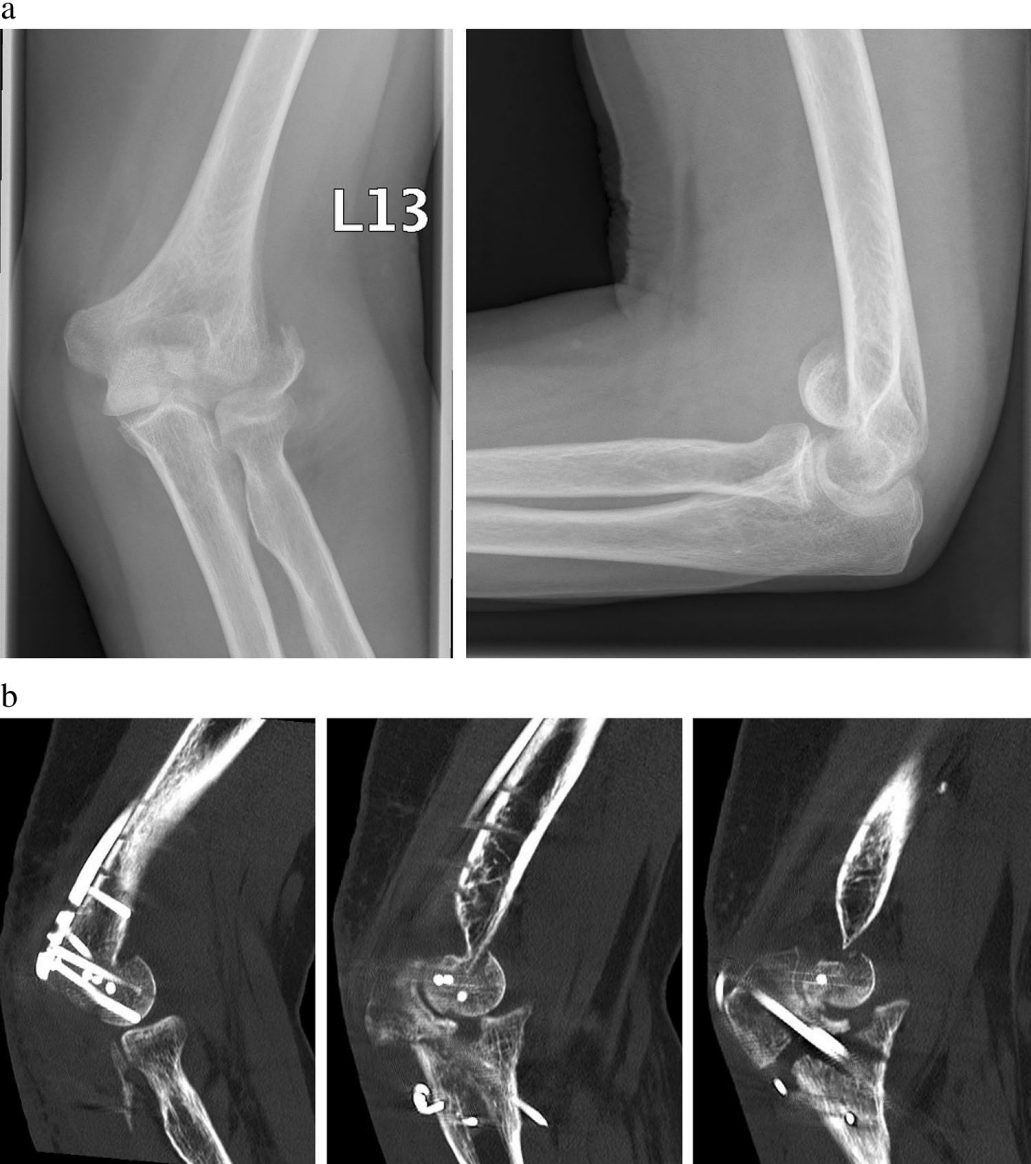
Case of a 63-year old female patient with a left-sided coronal shear fracture of the distal humerus (Dubberley type 3A) with concomitant fracture of the lateral condyle. She underwent ORIF with cannulated screws and a posterolateral distal humerus plate through a trans-olecranon approach. The patient presented to our office for the first time two months postoperatively due to limited range of motion of the affected elbow and sensory ulnar neuropathy. **A** Plain radiographs of the left elbow in an anteroposterior and lateral view following trauma show a displaced Dubberley type 3A fracture. **B** A CT scan of the left elbow two months postoperatively reveals insufficient reduction of the articular surface of the distal humerus. The olecranon osteotomy was performed too distal and significant incongruity of the articular surface of the proximal ulna remained following refixation with a tension band

While isolated fractures of the capitulum without relevant comminution (Bryan Morrey types 1 and 2, Dubberley type 1A) [10, 11] can be approached through an extended lateral approach, more invasive approaches have been propagated for complex fracture situations with posterior comminution and/or involvement of the trochlea to aid in visualization of the articular surface [1, 5, 12]. It is our clinical experience, however, that more extensile approaches can be avoided in a significant amount of cases. Especially the trans-olecranon approach with osteotomy of the olecranon—which is the most commonly used approach for comminuted type C fractures of the distal humerus—is not necessarily helpful in coronal shear fractures as it provides limited access to the anterior aspect of the articular cartilage (Fig. 1) [13].

Concomitant (epi-)condyle fractures have been reported in approximately one third of patients with coronal shear fractures of the distal humerus [2, 14]. These accompanying fractures can be used for a trans-fracture approach to improve visualization without increasing the approach-related morbidity. A lateral condyle fracture presents a typical associated lesion in coronal shear fractures of the distal humerus and can be helpful to improve visualization as it allows joint subluxation without harming the lateral collateral ligament complex and the common extensors which originate at this fragment.

Therefore, this retrospective study aimed to investigate the frequency of lateral condyle fractures in coronal shear fractures of the distal humerus and to analyze fracture reduction, fracture union and clinical results following open reduction and internal fixation (ORIF) through a trans-fracture approach.

## Materials and methods

A retrospective chart review of a 3-year period (2017–2019) was performed to identify all adult patients (age ≥ 18 years) who underwent treatment for an acute distal humerus fracture at our level-one trauma center.

Age, sex and the affected side of the patients were obtained. Open fractures were classified according to the unified classification of open fractures of Agrawal [15]. Fracture morphology was classified according to the Orthopaedic Trauma Association (OTA/AO) fracture classification system [16]. All B3 fractures were subclassified according to the classification proposed by Dubberley et al. [10]. The primary treatment (ORIF, arthroplasty, conservative) was obtained and all coronal shear fractures with concomitant lateral condyle fractures who underwent ORIF through a trans-fracture approach were further evaluated. Fracture reduction and fracture union were assessed by follow-up CT scans. Clinical results according to the Mayo Elbow Performance Score, the Visual Analogue Scale (VAS; pain level from 0to10; 0 = no pain; 10 = strongest pain imaginable) and the active range of motion were investigated. Complications and revision surgeries were noted.

## Results

### Overall results of distal humerus fractures

53 adult patients with an acute distal humerus fracture were identified. The mean age of patients was 52 ± 19 years (range: 18–87 years). 50.9% of patients were female, 49.1% were male. 60.4% were right-sided injuries, in 39.6% the left side was affected.

**Fig. 2:**
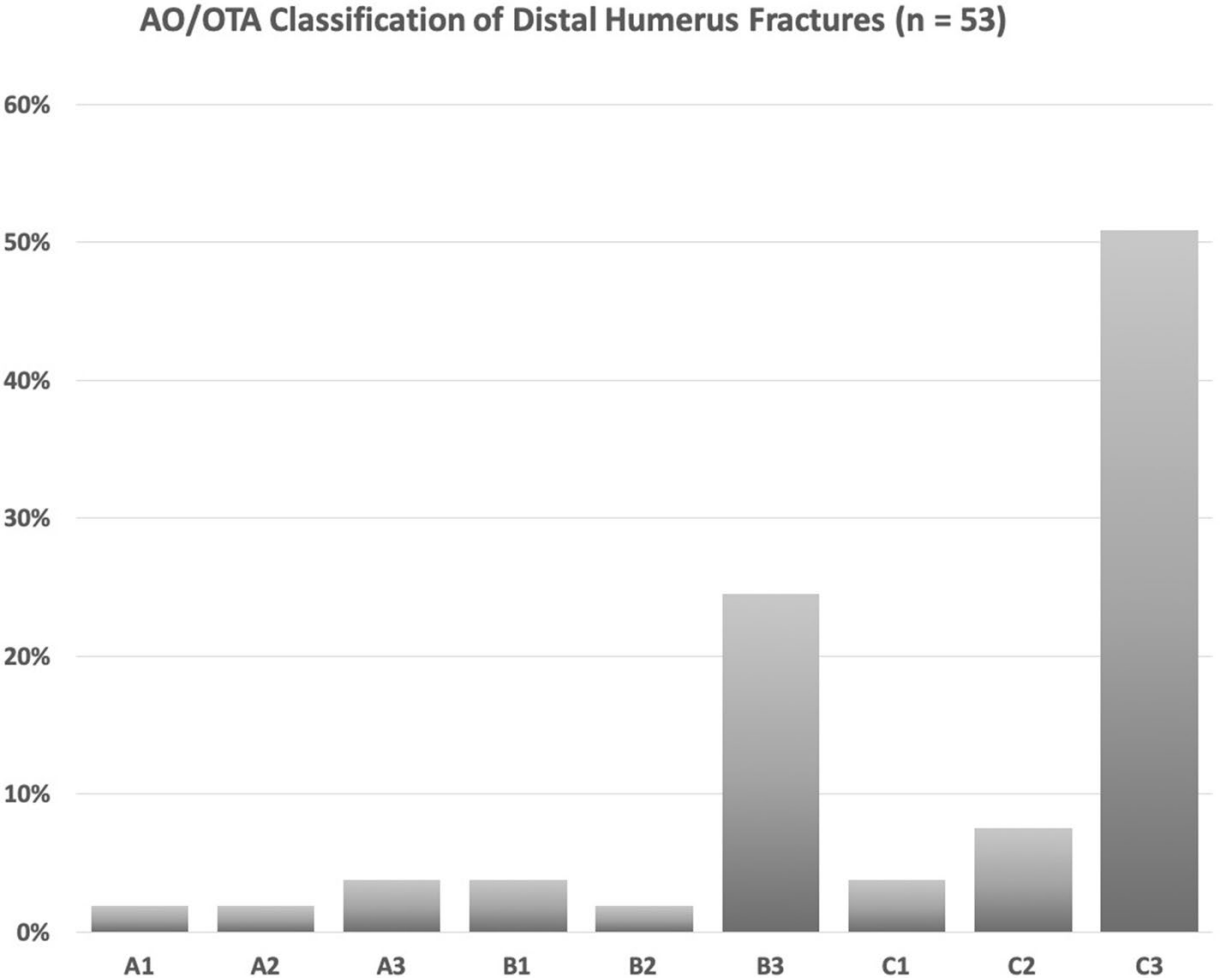
Fracture classification according to the Orthopaedic Trauma Association (OTA/AO) fracture classification system [16] (*n* = 53)

**Fig. 3:**
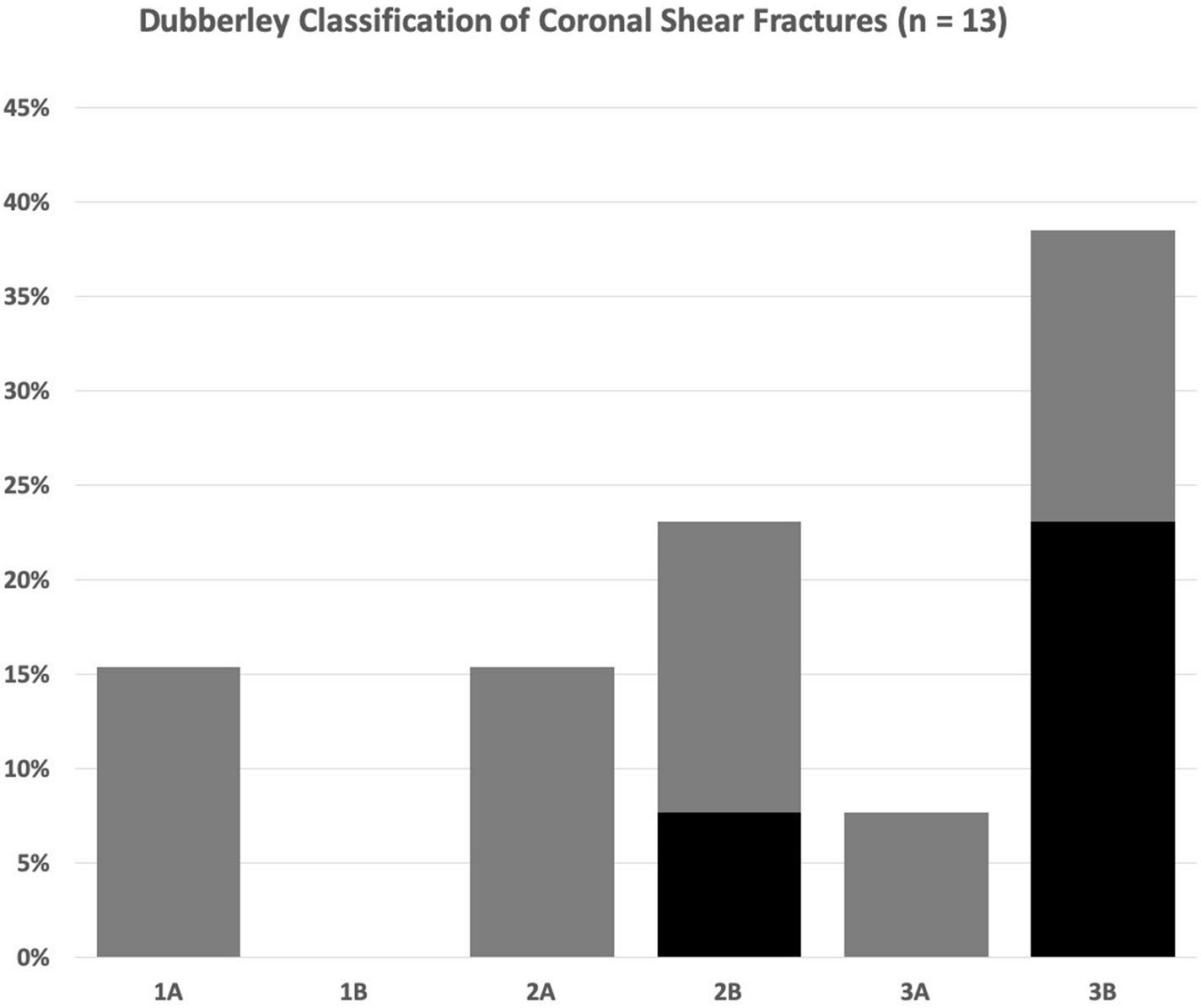
Classification of coronal shear fractures according to Dubberley et al. [10] 30.8% of patients with coronal shear fractures had an additional radial condyle fracture (black columns)

More than half of the fractures were classified as C3 fractures; approximately one fourth were B3 fractures. Two fractures were open fractures (3.8%); one type I and one type II according to the unified classification of open fractures of Agrawal [15]. Figures 2 and 3 outline the results for fracture classification according to the OTA/AO fracture classification system and according to the classification of Dubberley et al. [10, 16]

Most patients were treated with ORIF (75.5%; mean age: 45 ± 16 years); in one of these patients, reduction and screw fixation was performed arthroscopically. Seven low-demand patients (13.2%) with a mean age of 79 ± 5 years underwent primary total elbow arthroplasty; in four patients, hemiarthroplasty was performed (7.5%; mean age: 64 ± 5 years). Two patients were treated conservatively (3.8%).

### Results of coronal shear fractures with concomitant radial condyle fractures

**Fig. 4:**
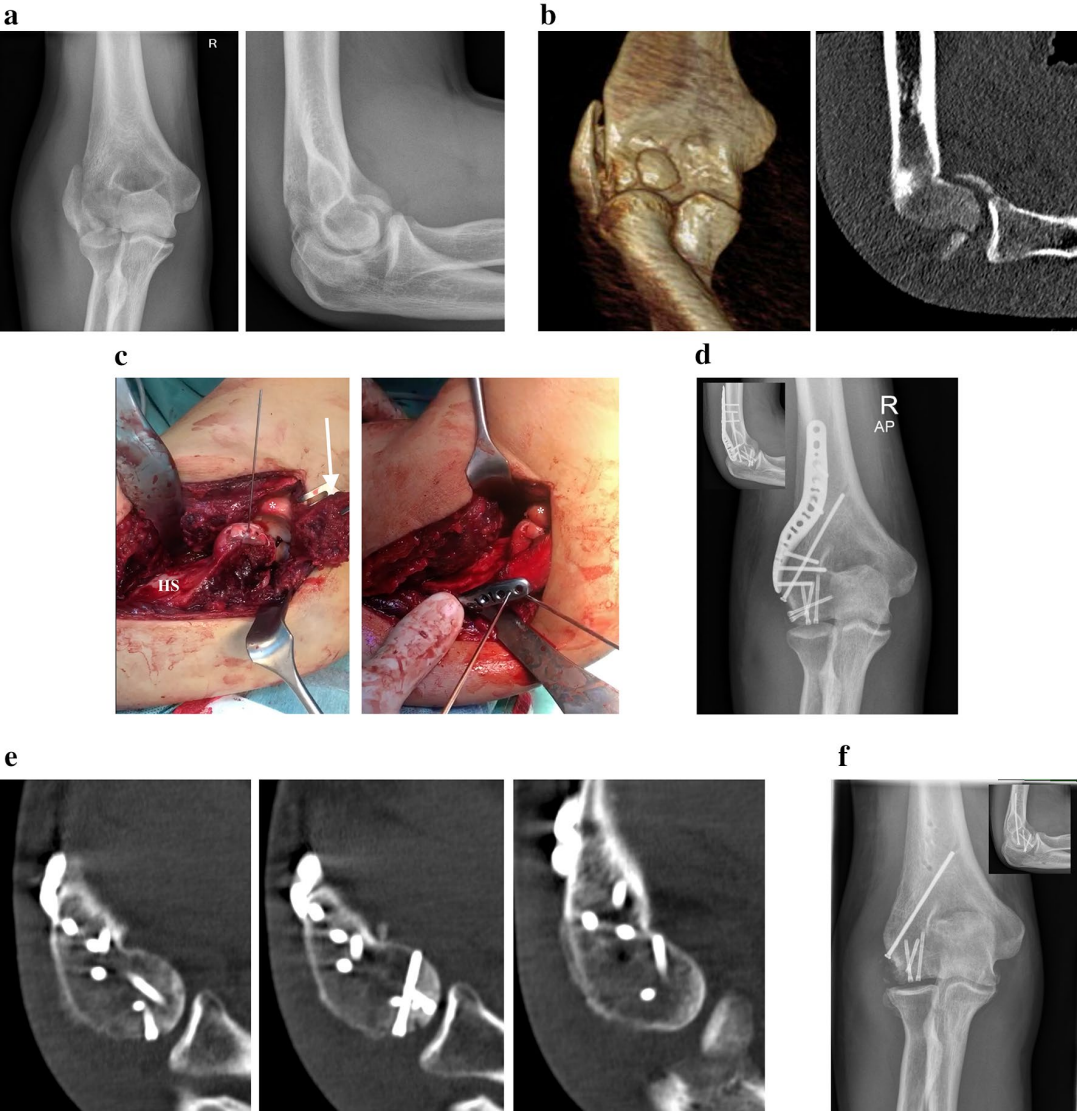
Case of a 32-year old male patient with a comminuted capitulum fracture (Dubberley type 2B) with concomitant radial condyle fracture of the right elbow. **A** Plain radiographs preoperatively. **B** Preoperative CT scan. **C** Intraoperative view. Following reflection of the radial condyle (white arrow), the articular surface can be restored. The radial condyle is then reduced and internal fixation with a precontoured lateral distal humerus locking plate is performed. **D** Plain radiographs postoperatively. **E** CT scan at 3 months to confirm anatomic reduction and fracture union. **F** Plain radiographs following partial implant removal and ligament bracing of the LUCL

**Fig. 5:**
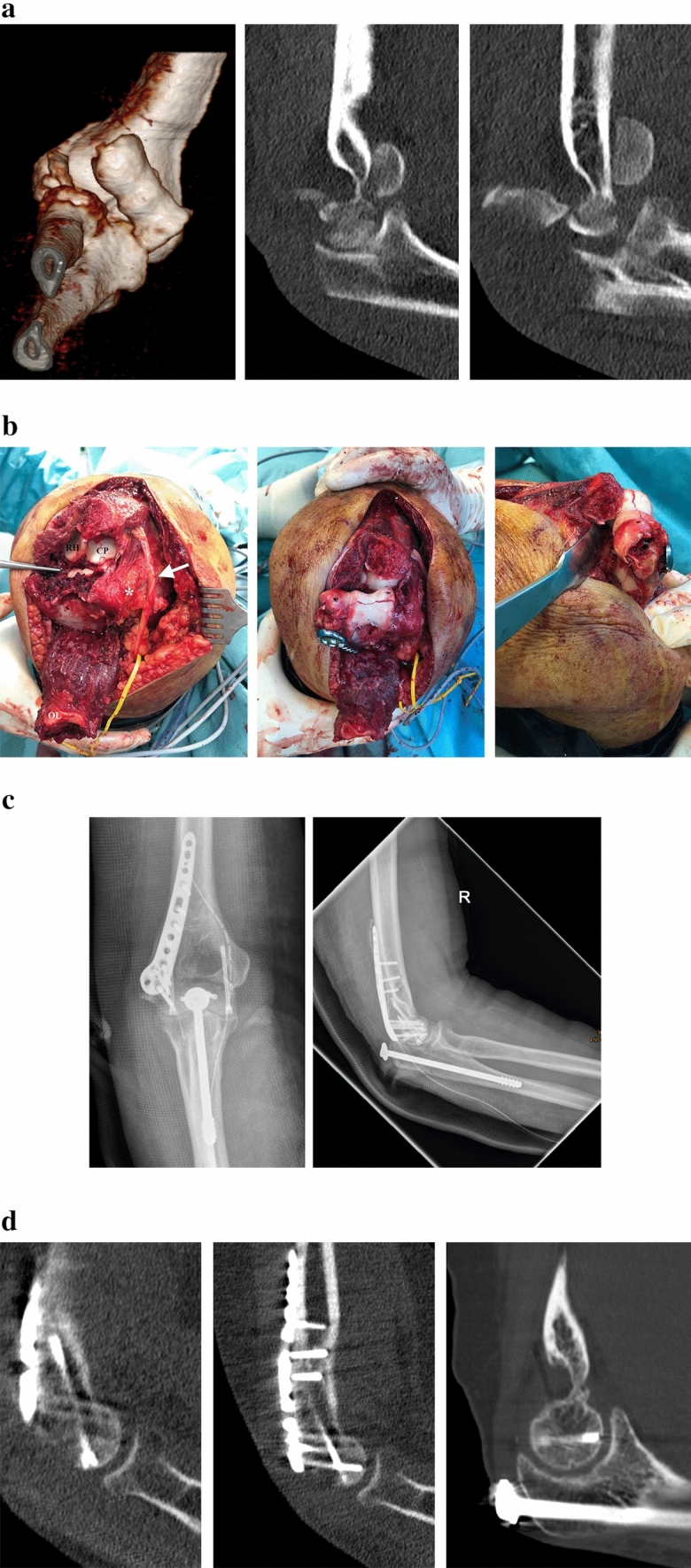
Case of a 53-year old female patient with a Dubberley type 3B fracture with concomitant radial condyle fracture of the right elbow. **A** Preoperative CT scan. **B** Intraoperative view. Using the radial condyle and olecranon fractures, a trans-fracture approach was applied to achieve ORIF while sparing the ulnar collateral ligament and the common flexor origin (*). The forceps points to the displaced articular surface. OL = Olecranon fragment; RH = Radial head; CP = Coronoid process; White arrow = Transposed ulnar nerve. **C** Postoperative plain radiographs. **D** Follow-up CT scan at one year revealing anatomic reduction and fracture union

**Fig. 6:**
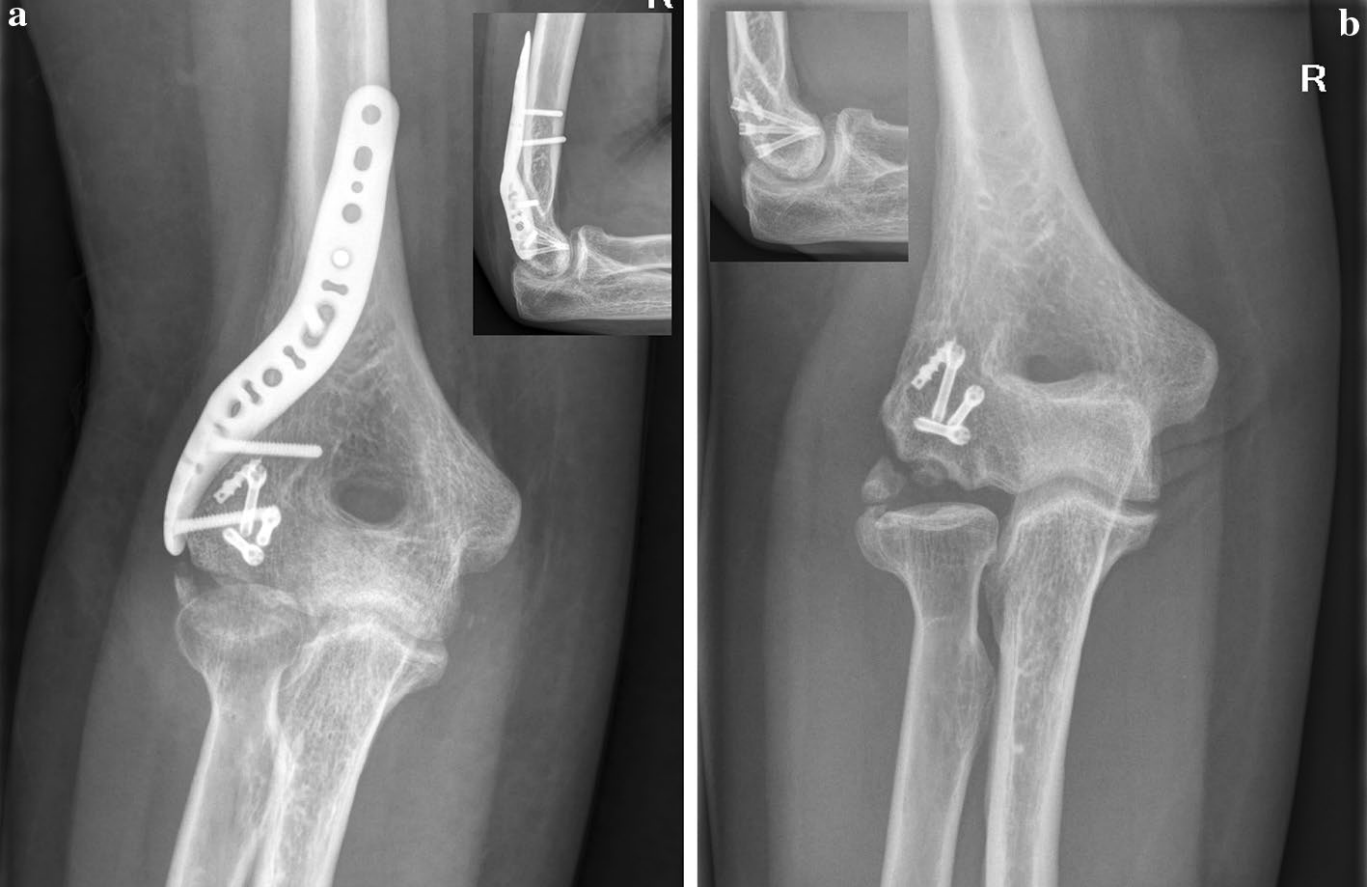
Case of a 33-year old female patient who developed partial avascular necrosis. **A** Plain radiographs 3 months postoperatively. **B** Plain radiographs following partial implant removal and arthrolysis

Overall, four patients (7.5% of all patients; 30.8% of patients with coronal shear fractures) had a coronal shear fracture with an additional radial condyle fracture. All of these patients underwent ORIF through a trans-fracture approach in a supine position. In three cases a lateral skin incision was used, in one case with an additional olecranon fracture a posterior skin incision was used. The lateral condyle fracture was used to reflect the lateral collateral ligament and the common extensor tendons, which allowed for joint subluxation. Open reduction of the articular surface was performed with headless cannulated compression screws (APTUS^®^ SpeedTip^®^ 2.2/3.0 mm, Medartis, Basel, CH). Refixation of the lateral condyle was achieved with an anatomic locking plate. Depending on fracture morphology, a lateral or posterolateral plate was used (Aptus^®^ Elbow System, 2.8 mm, Medartis, Basel, CH). In one patient with a concomitant olecranon fracture, additional ORIF of the olecranon was performed with an intramedullary compression screw (Figs. 4, 5, 6).

At a minimum follow-up of 24 months (24–27 months), the mean Mayo Elbow Performance Score was 88 ± 12 points (range: 70–100 points) with two excellent, one good and one fair result. The VAS was 2 ± 1 (range: 0–4). The active range of motion (flexion–extension arc) was 118° ± 12° (range: 105°–130°). All fractures showed anatomic reduction and three of four cases showed union within three months. One patient developed partial avascular necrosis of the capitulum. She underwent revision surgery with arthroscopic arthrolysis and plate removal at 6 months. This patient had a Mayo Elbow Performance Score of 70 points (fair result) and the VAS was 4. One patient underwent plate removal at 6 months and ligament bracing of the lateral ulnar collateral ligament (LUCL) was performed due to slight posterolateral rotatory instability. No further revision surgeries or complications occurred until the latest follow-up (Table 1).

**Table 1:** Patient data and clinical outcome of coronal shear fractures with concomitant radial

Case	Age (in y)	Sex	Side	Cx	Tx	FU (in mo)	MEPS	RoM	VAS	Compl	Revision surgery
#1	32	M	R	2B	CCS + LP	24	95	130°	0	PLRI	Implant removal + LUCL ligament bracing
#2	33	F	R	3B	CCS + LP	27	70	110°	4	Partial AN	Implant removal + arthrolysis
#3	53	F	R	3B	CCS + PLP + ORIF olecranon	24	100	125°	0	–	–
#4	56	F	R	3B	CCS + PLP	26	85	105°	2	–	–
*Mean*	*44*					*25*	*88*	*118°*	*2*		

Condyle fracture treated through a trans-fracture approach

*Y* years; *Cx* classification according to Dubberley et al. [10]; *mo* months; *RoM* Range of Motion; *VAS* visual analogue scale; *Compl* complications; *M* male; *F* female; *R* right; *CCS* cannulated compression screw; LP = anatomic lateral distal humerus locking plate; *PLP* anatomic posterolateral distal humerus locking plate; *ORIF* open reduction and internal fixation; *PLRI* posterolateral rotatory instability; *LUCL* lateral ulnar collateral ligament; *AN* avascular necrosis

## Discussion

The present study investigated the frequency of lateral condyle fractures in coronal shear fractures of the distal humerus and analyzed fracture reduction, fracture union and clinical short-term results following open reduction and internal fixation through a trans-fracture approach. The results of this investigation show that concomitant lateral condyle fractures are present in up to one third of coronal shear fractures of the distal humerus and that a trans-fracture approach is useful to reliably achieve anatomic reduction of these fractures. While the clinical outcome is generally favorable, avascular necrosis remains an issue limiting the long-term prognosis.

Coronal shear fractures of the distal humerus are challenging injuries and their treatment options are equally diverse and complex. While fragment excision leads to poor results, [17] some authors have reported good outcomes following closed reduction of displaced capitellar fractures [18–21]. Both Cutbuch et al. [18] and Puloski et al. [21] published seven cases of patients with displaced type 1 fractures of the capitulum (two-part fractures without comminution) according to the modified Bryan/Morrey classification [3] who were successfully treated with closed reduction. Fracture reduction was achieved under general anesthesia by extending the elbow in full supination, applying gentle traction, varus load and/or manual pressure anteriorly on the fragment with the thumb. The elbow was then flexed to 90° to trap the fragment between the radial head and the intact capitulum. All fractures healed in anatomic or near-anatomic position and a good clinical outcome was reported in 13 of 14 patients; one patient developed a flexion contracture of 45° [18, 21]. When closed reduction cannot be achieved, arthroscopic reduction and screw fixation may be a viable, minimally-invasive treatment modality for non-comminuted fractures and was feasible in one case in the present study. Clinical data on arthroscopically assisted reduction and fixation of coronal shear fractures of the distal humerus is, however, sparse and limited to case reports thus far [22–24].

If the fracture is not reconstructable due to severe comminution, hemiarthroplasty or total elbow arthroplasty might be considered [25, 26]. The majority of displaced and comminuted fractures, as shown in the present investigation, are amenable to open reduction and internal fixation. An extended lateral Kocher or Kaplan approach has uniformly been recommended by many authors for coronal shear fractures of the capitulum and the lateral aspect of the trochlea [1–6, 8, 10, 27–29]. In more complex fracture patterns involving the entirety of the trochlea and/or with presence of posterior comminution, however, some discrepancy exists in the literature regarding the most suitable approach. While some authors recommend to release the lateral collateral ligament complex off of the distal humerus to allow subluxation of the joint and thereby improving the exposure of the articular surface, [3, 10, 28] others prefer a bilateral approach by establishing an additional medial window through a flexor carpi ulnaris split or a Hotchkiss approach [30] to gain the required exposure without sacrificing crucial ligamentous stabilizers [12, 14]. Some authors even propagate a trans-olecranon approach with osteotomy of the olecranon [1, 5, 10]. This approach is of great help when treating comminuted C3 type fractures but, in our experience, is of little value to provide the required exposure of the anterior aspect of the articular surface to treat complex coronal shear fractures.

Notably, many authors have described associated fractures of the (epi-)condyles in up to one third of patients with coronal shear fractures [2, 14]. Especially a fractured radial condyle is frequently observed in these injuries as confirmed in the present study. This injury allows to reflect the lateral collateral ligament complex along with the common extensor origin and thereby provides excellent exposure of the articular surface without increasing the approach-related morbidity. As a result, anatomic fracture reduction was feasible in all cases without the need of an additional osteotomy or collateral ligament release. While this work reports on a small number of cases and does not provide any comparative data to other approaches mentioned before, it should make aware to look for these concomitant injuries when dealing with coronal shear fractures of the distal humerus as this might facilitate fracture reduction and fixation.

## Conclusions

To conclude, fractures of the lateral condyle are frequent concomitant injuries of coronal shear fractures of the distal humerus. They are observed in close to one third of all cases. The presence of this concomitant injury can be used for a trans-fracture approach to facilitate exposure and to reliably achieve anatomic fracture reduction without the need of sacrificing additional bony or ligamentous stabilizers. The functional outcomes following a trans-fracture approach through the radial condyle are promising, yet the available data are limited and the risk of avascular necrosis persists. Further research is necessary to investigate whether this approach may be able to improve the clinical results and lower the complication rates of coronal shear fractures of the distal humerus when compared to conventionally performed approaches.
